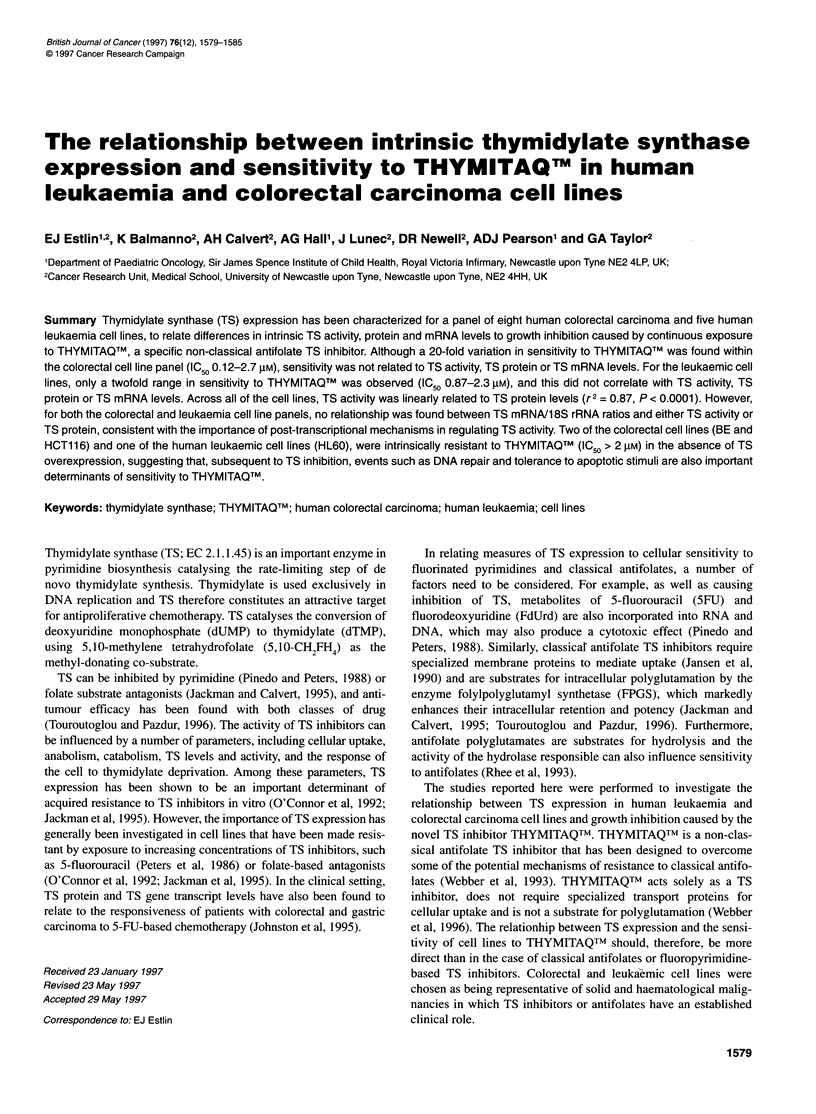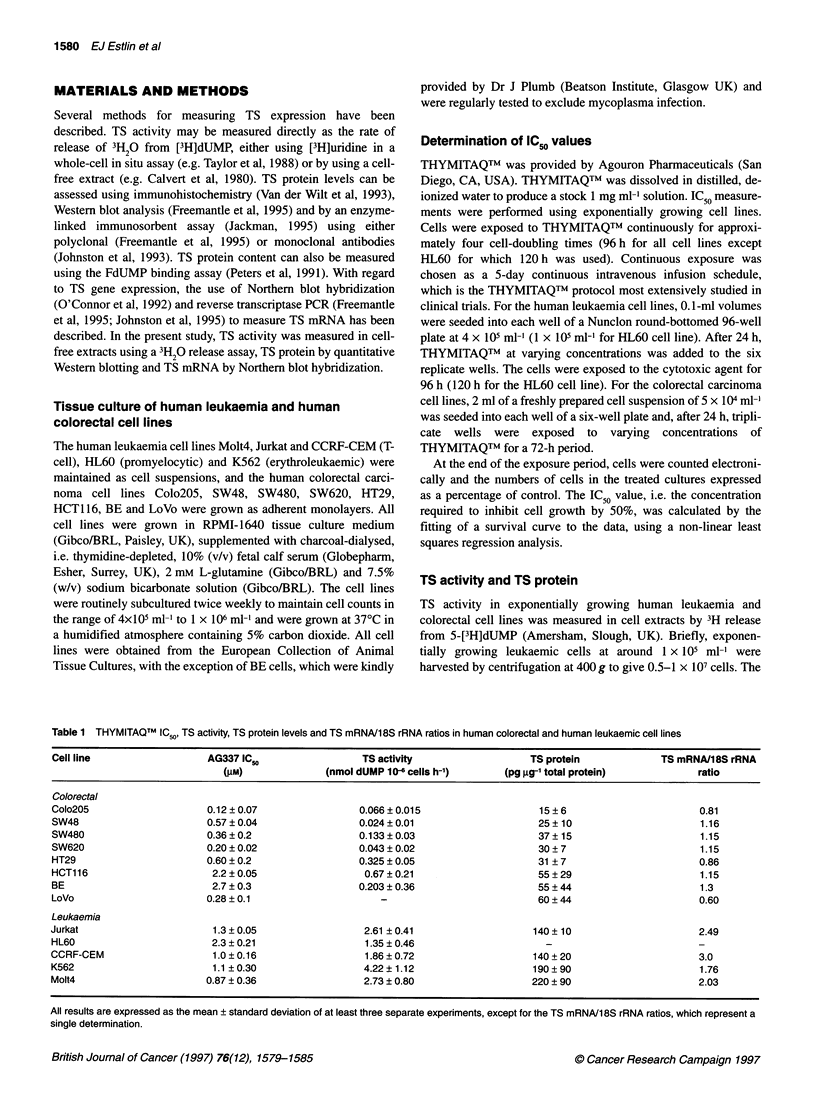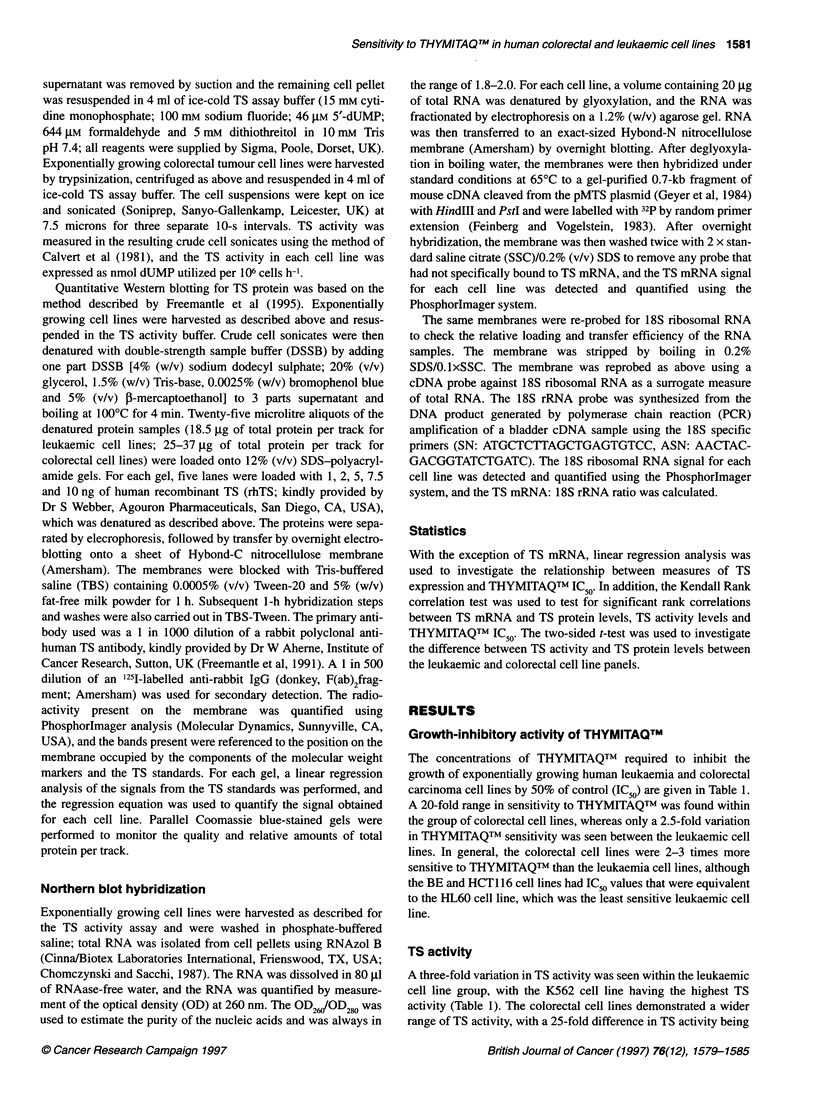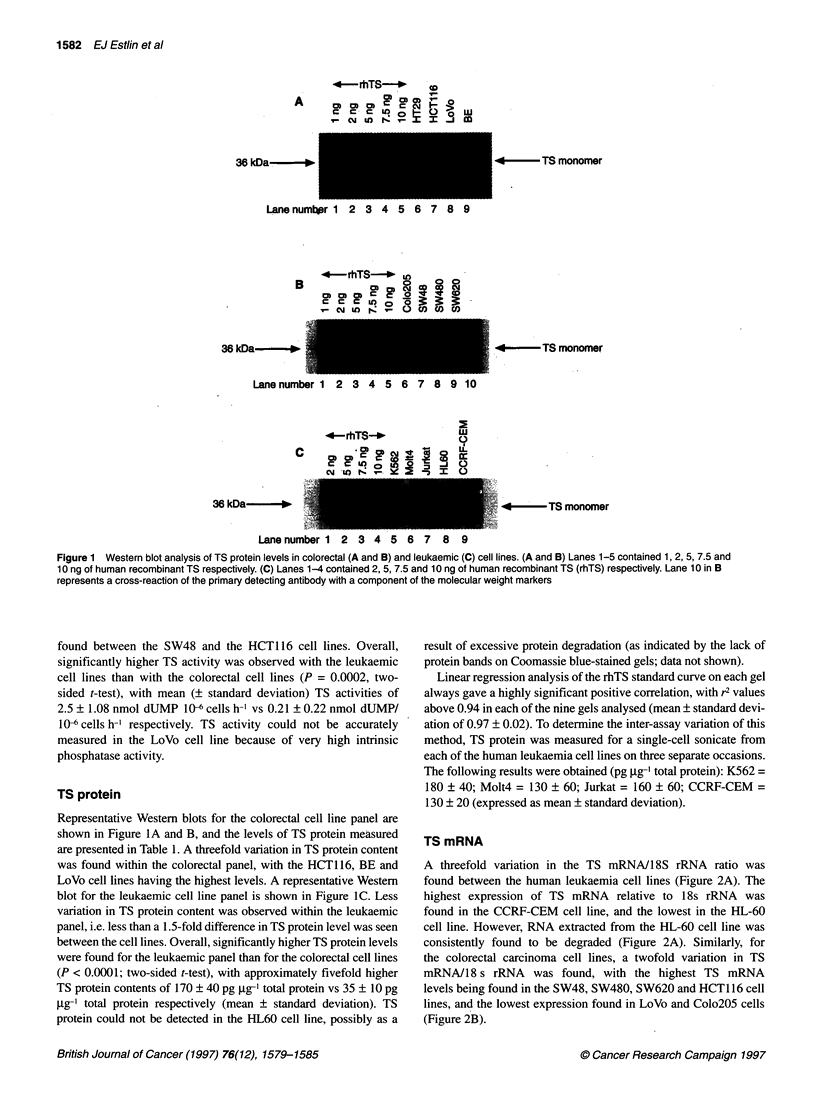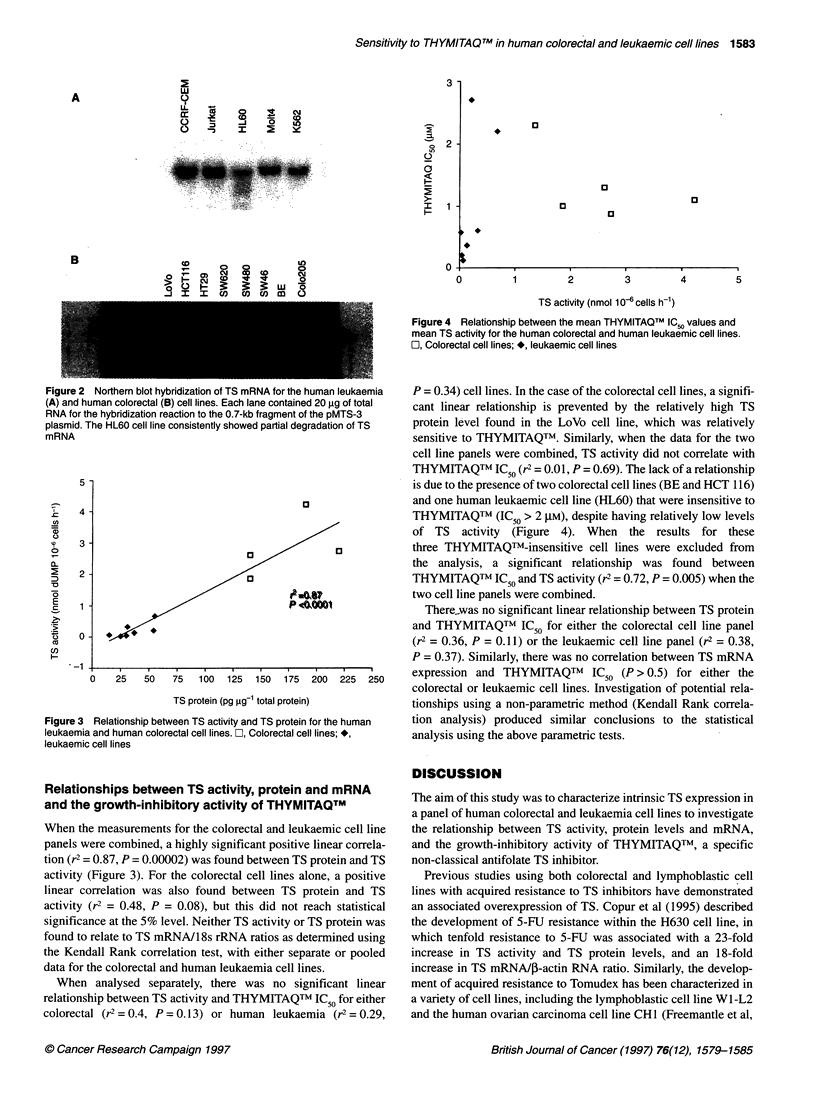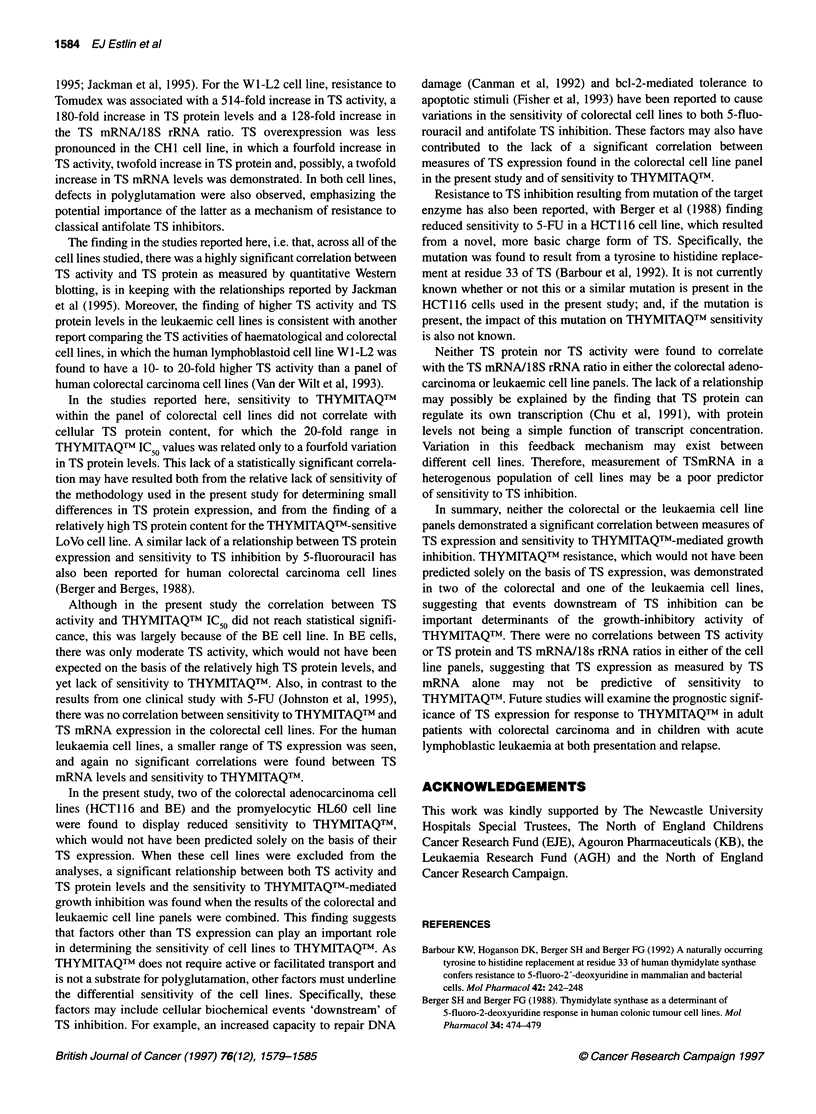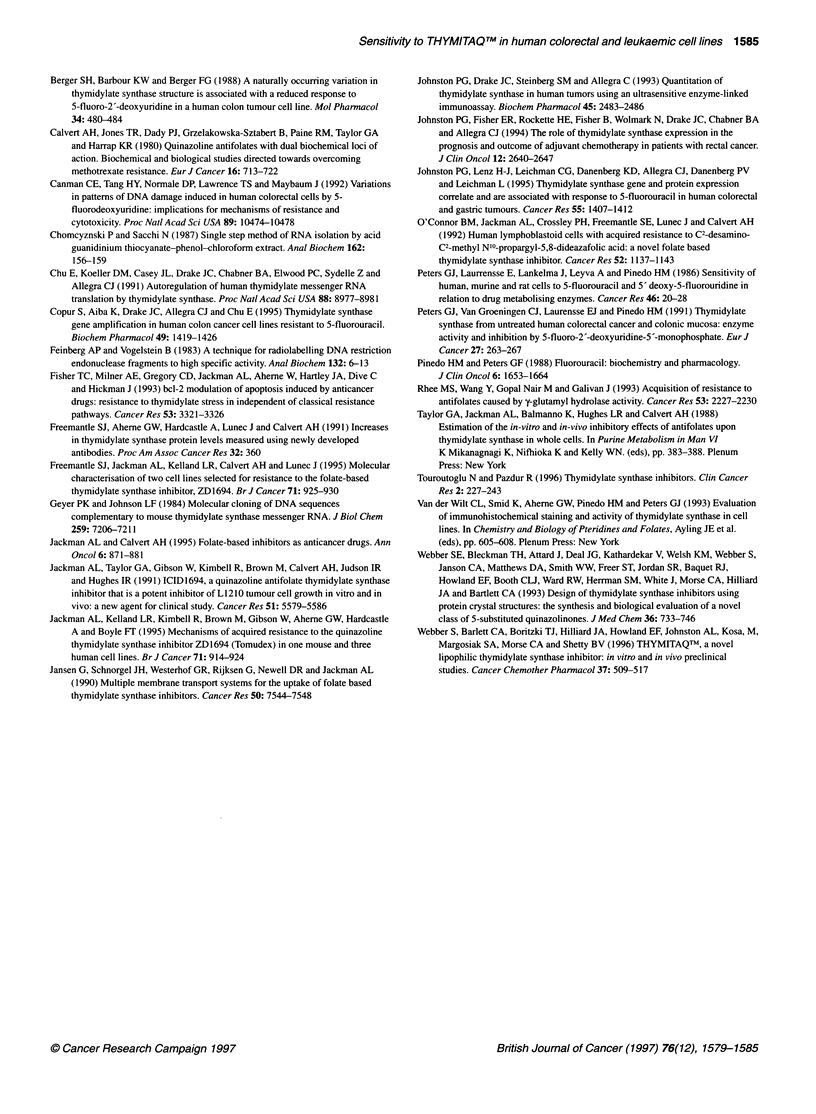# The relationship between intrinsic thymidylate synthase expression and sensitivity to THYMITAQ in human leukaemia and colorectal carcinoma cell lines.

**DOI:** 10.1038/bjc.1997.600

**Published:** 1997

**Authors:** E. J. Estlin, K. Balmanno, A. H. Calvert, A. G. Hall, J. Lunec, D. R. Newell, A. D. Pearson, G. A. Taylor

**Affiliations:** Department of Paediatric Oncology, Sir James Spence Institute of Child Health, Royal Victoria Infirmary, Newcastle upon Tyne, UK.

## Abstract

**Images:**


*British Journal of Cancer* (1997) **76**(12), 1579–1585
© 1997 Cancer Research Campaign

### The relationship between intrinsic thymidylate synthase expression and sensitivity to THYMITAQ™ in human leukaemia and colorectal carcinoma cell lines

**EJ Estlin[1,2], K Balmanno[2], AH Calvert[2], AG Hall[1], J Lunec[2], DR Newell[2], ADJ Pearson[1] and GA Taylor[2]**

[1]Department of Paediatric Oncology, Sir James Spence Institute of Child Health, Royal Victoria Infirmary, Newcastle upon Tyne NE2 4LP, UK;
[2]Cancer Research Unit, Medical School, University of Newcastle upon Tyne, Newcastle upon Tyne, NE2 4HH, UK

**Summary** Thymidylate synthase (TS) expression has been characterized for a panel of eight human colorectal carcinoma and five human leukaemia cell lines, to relate differences in intrinsic TS activity, protein and mRNA levels to growth inhibition caused by continuous exposure to THYMITAQ™, a specific non-classical antifolate TS inhibitor. Although a 20-fold variation in sensitivity to THYMITAQ™ was found within the colorectal cell line panel ($IC_{50}$ 0.12–2.7 μM), sensitivity was not related to TS activity, TS protein or TS mRNA levels. For the leukaemic cell lines, only a twofold range in sensitivity to THYMITAQ™ was observed ($IC_{50}$ 0.87–2.3 μM), and this did not correlate with TS activity, TS protein or TS mRNA levels. Across all of the cell lines, TS activity was linearly related to TS protein levels ($r^2 = 0.87$, $P < 0.0001$). However, for both the colorectal and leukaemia cell line panels, no relationship was found between TS mRNA/18S rRNA ratios and either TS activity or TS protein, consistent with the importance of post-transcriptional mechanisms in regulating TS activity. Two of the colorectal cell lines (BE and HCT116) and one of the human leukaemic cell lines (HL60), were intrinsically resistant to THYMITAQ™ ($IC_{50} > 2$ μM) in the absence of TS overexpression, suggesting that, subsequent to TS inhibition, events such as DNA repair and tolerance to apoptotic stimuli are also important determinants of sensitivity to THYMITAQ™.

**Keywords:** thymidylate synthase; THYMITAQ™; human colorectal carcinoma; human leukaemia; cell lines

Thymidylate synthase (TS; EC 2.1.1.45) is an important enzyme in pyrimidine biosynthesis catalysing the rate-limiting step of de novo thymidylate synthesis. Thymidylate is used exclusively in DNA replication and TS therefore constitutes an attractive target for antiproliferative chemotherapy. TS catalyses the conversion of deoxyuridine monophosphate (dUMP) to thymidylate (dTMP), using 5,10-methylene tetrahydrofolate (5,10-$CH_2FH_4$) as the methyl-donating co-substrate.

TS can be inhibited by pyrimidine (Pinedo and Peters, 1988) or folate substrate antagonists (Jackman and Calvert, 1995), and anti-tumour efficacy has been found with both classes of drug (Touroutoglou and Pazdur, 1996). The activity of TS inhibitors can be influenced by a number of parameters, including cellular uptake, anabolism, catabolism, TS levels and activity, and the response of the cell to thymidylate deprivation. Among these parameters, TS expression has been shown to be an important determinant of acquired resistance to TS inhibitors in vitro (O'Connor et al, 1992; Jackman et al, 1995). However, the importance of TS expression has generally been investigated in cell lines that have been made resistant by exposure to increasing concentrations of TS inhibitors, such as 5-fluorouracil (Peters et al, 1986) or folate-based antagonists (O'Connor et al, 1992; Jackman et al, 1995). In the clinical setting, TS protein and TS gene transcript levels have also been found to relate to the responsiveness of patients with colorectal and gastric carcinoma to 5-FU-based chemotherapy (Johnston et al, 1995).

*Received 23 January 1997*
*Revised 23 May 1997*
*Accepted 29 May 1997*

*Correspondence to:* EJ Estlin

In relating measures of TS expression to cellular sensitivity to fluorinated pyrimidines and classical antifolates, a number of factors need to be considered. For example, as well as causing inhibition of TS, metabolites of 5-fluorouracil (5FU) and fluorodeoxyuridine (FdUrd) are also incorporated into RNA and DNA, which may also produce a cytotoxic effect (Pinedo and Peters, 1988). Similarly, classical antifolate TS inhibitors require specialized membrane proteins to mediate uptake (Jansen et al, 1990) and are substrates for intracellular polyglutamation by the enzyme folylpolyglutamyl synthetase (FPGS), which markedly enhances their intracellular retention and potency (Jackman and Calvert, 1995; Touroutoglou and Pazdur, 1996). Furthermore, antifolate polyglutamates are substrates for hydrolysis and the activity of the hydrolase responsible can also influence sensitivity to antifolates (Rhee et al, 1993).

The studies reported here were performed to investigate the relationship between TS expression in human leukaemia and colorectal carcinoma cell lines and growth inhibition caused by the novel TS inhibitor THYMITAQ™. THYMITAQ™ is a non-classical antifolate TS inhibitor that has been designed to overcome some of the potential mechanisms of resistance to classical antifolates (Webber et al, 1993). THYMITAQ™ acts solely as a TS inhibitor, does not require specialized transport proteins for cellular uptake and is not a substrate for polyglutamation (Webber et al, 1996). The relationhip between TS expression and the sensitivity of cell lines to THYMITAQ™ should, therefore, be more direct than in the case of classical antifolates or fluoropyrimidine-based TS inhibitors. Colorectal and leukaemic cell lines were chosen as being representative of solid and haematological malignancies in which TS inhibitors or antifolates have an established clinical role.

### MATERIALS AND METHODS

Several methods for measuring TS expression have been described. TS activity may be measured directly as the rate of release of $^3H_2O$ from [$^3$H]dUMP, either using [$^3$H]uridine in a whole-cell in situ assay (e.g. Taylor et al, 1988) or by using a cell-free extract (e.g. Calvert et al, 1980). TS protein levels can be assessed using immunohistochemistry (Van der Wilt et al, 1993), Western blot analysis (Freemantle et al, 1995) and by an enzyme-linked immunosorbent assay (Jackman, 1995) using either polyclonal (Freemantle et al, 1995) or monoclonal antibodies (Johnston et al, 1993). TS protein content can also be measured using the FdUMP binding assay (Peters et al, 1991). With regard to TS gene expression, the use of Northern blot hybridization (O'Connor et al, 1992) and reverse transcriptase PCR (Freemantle et al, 1995; Johnston et al, 1995) to measure TS mRNA has been described. In the present study, TS activity was measured in cell-free extracts using a $^3H_2O$ release assay, TS protein by quantitative Western blotting and TS mRNA by Northern blot hybridization.

### Tissue culture of human leukaemia and human colorectal cell lines

The human leukaemia cell lines Molt4, Jurkat and CCRF-CEM (T-cell), HL60 (promyelocytic) and K562 (erythroleukaemic) were maintained as cell suspensions, and the human colorectal carcinoma cell lines Colo205, SW48, SW480, SW620, HT29, HCT116, BE and LoVo were grown as adherent monolayers. All cell lines were grown in RPMI-1640 tissue culture medium (Gibco/BRL, Paisley, UK), supplemented with charcoal-dialysed, i.e. thymidine-depleted, 10% (v/v) fetal calf serum (Globepharm, Esher, Surrey, UK), 2 mM L-glutamine (Gibco/BRL) and 7.5% (w/v) sodium bicarbonate solution (Gibco/BRL). The cell lines were routinely subcultured twice weekly to maintain cell counts in the range of $4 \times 10^5$ ml$^{-1}$ to $1 \times 10^6$ ml$^{-1}$ and were grown at 37°C in a humidified atmosphere containing 5% carbon dioxide. All cell lines were obtained from the European Collection of Animal Tissue Cultures, with the exception of BE cells, which were kindly provided by Dr J Plumb (Beatson Institute, Glasgow UK) and were regularly tested to exclude mycoplasma infection.

### Determination of $IC_{50}$ values

THYMITAQ™ was provided by Agouron Pharmaceuticals (San Diego, CA, USA). THYMITAQ™ was dissolved in distilled, deionized water to produce a stock 1 mg ml$^{-1}$ solution. $IC_{50}$ measurements were performed using exponentially growing cell lines. Cells were exposed to THYMITAQ™ continuously for approximately four cell-doubling times (96 h for all cell lines except HL60 for which 120 h was used). Continuous exposure was chosen as a 5-day continuous intravenous infusion schedule, which is the THYMITAQ™ protocol most extensively studied in clinical trials. For the human leukaemia cell lines, 0.1-ml volumes were seeded into each well of a Nunclon round-bottomed 96-well plate at $4 \times 10^5$ ml$^{-1}$ ($1 \times 10^5$ ml$^{-1}$ for HL60 cell line). After 24 h, THYMITAQ™ at varying concentrations was added to the six replicate wells. The cells were exposed to the cytotoxic agent for 96 h (120 h for the HL60 cell line). For the colorectal carcinoma cell lines, 2 ml of a freshly prepared cell suspension of $5 \times 10^4$ ml$^{-1}$ was seeded into each well of a six-well plate and, after 24 h, triplicate wells were exposed to varying concentrations of THYMITAQ™ for a 72-h period.

At the end of the exposure period, cells were counted electronically and the numbers of cells in the treated cultures expressed as a percentage of control. The $IC_{50}$ value, i.e. the concentration required to inhibit cell growth by 50%, was calculated by the fitting of a survival curve to the data, using a non-linear least squares regression analysis.

### TS activity and TS protein

TS activity in exponentially growing human leukaemia and colorectal cell lines was measured in cell extracts by $^3$H release from 5-[$^3$H]dUMP (Amersham, Slough, UK). Briefly, exponentially growing leukaemic cells at around $1 \times 10^5$ ml$^{-1}$ were harvested by centrifugation at 400 *g* to give 0.5–1 $\times 10^7$ cells. The

**Table 1** THYMITAQ™ $IC_{50}$, TS activity, TS protein levels and TS mRNA/18S rRNA ratios in human colorectal and human leukaemic cell lines

| Cell line | AG337 $IC_{50}$ (μM) | TS activity (nmol dUMP $10^{-6}$ cells h$^{-1}$) | TS protein (pg μg$^{-1}$ total protein) | TS mRNA/18S rRNA ratio |
|---|---|---|---|---|
| *Colorectal* | | | | |
| Colo205 | 0.12 ± 0.07 | 0.066 ± 0.015 | 15 ± 6 | 0.81 |
| SW48 | 0.57 ± 0.04 | 0.024 ± 0.01 | 25 ± 10 | 1.16 |
| SW480 | 0.36 ± 0.2 | 0.133 ± 0.03 | 37 ± 15 | 1.15 |
| SW620 | 0.20 ± 0.02 | 0.043 ± 0.02 | 30 ± 7 | 1.15 |
| HT29 | 0.60 ± 0.2 | 0.325 ± 0.05 | 31 ± 7 | 0.86 |
| HCT116 | 2.2 ± 0.05 | 0.67 ± 0.21 | 55 ± 29 | 1.15 |
| BE | 2.7 ± 0.3 | 0.203 ± 0.36 | 55 ± 44 | 1.3 |
| LoVo | 0.28 ± 0.1 | – | 60 ± 44 | 0.60 |
| *Leukaemia* | | | | |
| Jurkat | 1.3 ± 0.05 | 2.61 ± 0.41 | 140 ± 10 | 2.49 |
| HL60 | 2.3 ± 0.21 | 1.35 ± 0.46 | – | – |
| CCRF-CEM | 1.0 ± 0.16 | 1.86 ± 0.72 | 140 ± 20 | 3.0 |
| K562 | 1.1 ± 0.30 | 4.22 ± 1.12 | 190 ± 90 | 1.76 |
| Molt4 | 0.87 ± 0.36 | 2.73 ± 0.80 | 220 ± 90 | 2.03 |

All results are expressed as the mean ± standard deviation of at least three separate experiments, except for the TS mRNA/18S rRNA ratios, which represent a single determination.

© Cancer Research Campaign 1997

supernatant was removed by suction and the remaining cell pellet was resuspended in 4 ml of ice-cold TS assay buffer (15 mM cytidine monophosphate; 100 mM sodium fluoride; 46 μM 5′-dUMP; 644 μM formaldehyde and 5 mM dithiothreitol in 10 mM Tris pH 7.4; all reagents were supplied by Sigma, Poole, Dorset, UK). Exponentially growing colorectal tumour cell lines were harvested by trypsinization, centrifuged as above and resuspended in 4 ml of ice-cold TS assay buffer. The cell suspensions were kept on ice and sonicated (Soniprep, Sanyo-Gallenkamp, Leicester, UK) at 7.5 microns for three separate 10-s intervals. TS activity was measured in the resulting crude cell sonicates using the method of Calvert et al (1981), and the TS activity in each cell line was expressed as nmol dUMP utilized per $10^6$ cells $h^{-1}$.

Quantitative Western blotting for TS protein was based on the method described by Freemantle et al (1995). Exponentially growing cell lines were harvested as described above and resuspended in the TS activity buffer. Crude cell sonicates were then denatured with double-strength sample buffer (DSSB) by adding one part DSSB [4% (w/v) sodium dodecyl sulphate; 20% (v/v) glycerol, 1.5% (w/v) Tris-base, 0.0025% (w/v) bromophenol blue and 5% (v/v) β-mercaptoethanol] to 3 parts supernatant and boiling at 100°C for 4 min. Twenty-five microlitre aliquots of the denatured protein samples (18.5 μg of total protein per track for leukaemic cell lines; 25–37 μg of total protein per track for colorectal cell lines) were loaded onto 12% (v/v) SDS–polyacrylamide gels. For each gel, five lanes were loaded with 1, 2, 5, 7.5 and 10 ng of human recombinant TS (rhTS; kindly provided by Dr S Webber, Agouron Pharmaceuticals, San Diego, CA, USA), which was denatured as described above. The proteins were separated by elecrophoresis, followed by transfer by overnight electroblotting onto a sheet of Hybond-C nitrocellulose membrane (Amersham). The membranes were blocked with Tris-buffered saline (TBS) containing 0.0005% (v/v) Tween-20 and 5% (w/v) fat-free milk powder for 1 h. Subsequent 1-h hybridization steps and washes were also carried out in TBS-Tween. The primary antibody used was a 1 in 1000 dilution of a rabbit polyclonal anti-human TS antibody, kindly provided by Dr W Aherne, Institute of Cancer Research, Sutton, UK (Freemantle et al, 1991). A 1 in 500 dilution of an $^{125}I$-labelled anti-rabbit IgG (donkey, $F(ab)_2$fragment; Amersham) was used for secondary detection. The radioactivity present on the membrane was quantified using PhosphorImager analysis (Molecular Dynamics, Sunnyville, CA, USA), and the bands present were referenced to the position on the membrane occupied by the components of the molecular weight markers and the TS standards. For each gel, a linear regression analysis of the signals from the TS standards was performed, and the regression equation was used to quantify the signal obtained for each cell line. Parallel Coomassie blue-stained gels were performed to monitor the quality and relative amounts of total protein per track.

### Northern blot hybridization

Exponentially growing cell lines were harvested as described for the TS activity assay and were washed in phosphate-buffered saline; total RNA was isolated from cell pellets using RNAzol B (Cinna/Biotex Laboratories International, Frienswood, TX, USA; Chomczynski and Sacchi, 1987). The RNA was dissolved in 80 μl of RNAase-free water, and the RNA was quantified by measurement of the optical density (OD) at 260 nm. The $OD_{260}/OD_{280}$ was used to estimate the purity of the nucleic acids and was always in the range of 1.8–2.0. For each cell line, a volume containing 20 μg of total RNA was denatured by glyoxylation, and the RNA was fractionated by electrophoresis on a 1.2% (w/v) agarose gel. RNA was then transferred to an exact-sized Hybond-N nitrocellulose membrane (Amersham) by overnight blotting. After deglyoxylation in boiling water, the membranes were then hybridized under standard conditions at 65°C to a gel-purified 0.7-kb fragment of mouse cDNA cleaved from the pMTS plasmid (Geyer et al, 1984) with *Hin*dIII and *Pst*I and were labelled with $^{32}P$ by random primer extension (Feinberg and Vogelstein, 1983). After overnight hybridization, the membrane was then washed twice with 2 × standard saline citrate (SSC)/0.2% (v/v) SDS to remove any probe that had not specifically bound to TS mRNA, and the TS mRNA signal for each cell line was detected and quantified using the PhosphorImager system.

The same membranes were re-probed for 18S ribosomal RNA to check the relative loading and transfer efficiency of the RNA samples. The membrane was stripped by boiling in 0.2% SDS/0.1×SSC. The membrane was reprobed as above using a cDNA probe against 18S ribosomal RNA as a surrogate measure of total RNA. The 18S rRNA probe was synthesized from the DNA product generated by polymerase chain reaction (PCR) amplification of a bladder cDNA sample using the 18S specific primers (SN: ATGCTCTTAGCTGAGTGTCC, ASN: AACTACGACGGTATCTGATC). The 18S ribosomal RNA signal for each cell line was detected and quantified using the PhosphorImager system, and the TS mRNA: 18S rRNA ratio was calculated.

### Statistics

With the exception of TS mRNA, linear regression analysis was used to investigate the relationship between measures of TS expression and THYMITAQ™ $IC_{50}$. In addition, the Kendall Rank correlation test was used to test for significant rank correlations between TS mRNA and TS protein levels, TS activity levels and THYMITAQ™ $IC_{50}$. The two-sided *t*-test was used to investigate the difference between TS activity and TS protein levels between the leukaemic and colorectal cell line panels.

### RESULTS

### Growth-inhibitory activity of THYMITAQ™

The concentrations of THYMITAQ™ required to inhibit the growth of exponentially growing human leukaemia and colorectal carcinoma cell lines by 50% of control ($IC_{50}$) are given in Table 1. A 20-fold range in sensitivity to THYMITAQ™ was found within the group of colorectal cell lines, whereas only a 2.5-fold variation in THYMITAQ™ sensitivity was seen between the leukaemic cell lines. In general, the colorectal cell lines were 2–3 times more sensitive to THYMITAQ™ than the leukaemia cell lines, although the BE and HCT116 cell lines had $IC_{50}$ values that were equivalent to the HL60 cell line, which was the least sensitive leukaemic cell line.

### TS activity

A three-fold variation in TS activity was seen within the leukaemic cell line group, with the K562 cell line having the highest TS activity (Table 1). The colorectal cell lines demonstrated a wider range of TS activity, with a 25-fold difference in TS activity being

© Cancer Research Campaign 1997

**Figure 1** Western blot analysis of TS protein levels in colorectal (**A** and **B**) and leukaemic (**C**) cell lines. (**A** and **B**) Lanes 1–5 contained 1, 2, 5, 7.5 and 10 ng of human recombinant TS respectively. (**C**) Lanes 1–4 contained 2, 5, 7.5 and 10 ng of human recombinant TS (rhTS) respectively. Lane 10 in **B** represents a cross-reaction of the primary detecting antibody with a component of the molecular weight markers found between the SW48 and the HCT116 cell lines. Overall, significantly higher TS activity was observed with the leukaemic cell lines than with the colorectal cell lines ($P$ = 0.0002, two-sided $t$-test), with mean (± standard deviation) TS activities of 2.5 ± 1.08 nmol dUMP $10^{-6}$ cells $h^{-1}$ vs 0.21 ± 0.22 nmol dUMP/$10^{-6}$ cells $h^{-1}$ respectively. TS activity could not be accurately measured in the LoVo cell line because of very high intrinsic phosphatase activity.

### TS protein

Representative Western blots for the colorectal cell line panel are shown in Figure 1A and B, and the levels of TS protein measured are presented in Table 1. A threefold variation in TS protein content was found within the colorectal panel, with the HCT116, BE and LoVo cell lines having the highest levels. A representative Western blot for the leukaemic cell line panel is shown in Figure 1C. Less variation in TS protein content was observed within the leukaemic panel, i.e. less than a 1.5-fold difference in TS protein level was seen between the cell lines. Overall, significantly higher TS protein levels were found for the leukaemic panel than for the colorectal cell lines ($P < 0.0001$; two-sided $t$-test), with approximately fivefold higher TS protein contents of 170 ± 40 pg $\mu g^{-1}$ total protein vs 35 ± 10 pg $\mu g^{-1}$ total protein respectively (mean ± standard deviation). TS protein could not be detected in the HL60 cell line, possibly as a result of excessive protein degradation (as indicated by the lack of protein bands on Coomassie blue-stained gels; data not shown).

Linear regression analysis of the rhTS standard curve on each gel always gave a highly significant positive correlation, with $r^2$ values above 0.94 in each of the nine gels analysed (mean ± standard deviation of 0.97 ± 0.02). To determine the inter-assay variation of this method, TS protein was measured for a single-cell sonicate from each of the human leukaemia cell lines on three separate occasions. The following results were obtained (pg $\mu g^{-1}$ total protein): K562 = 180 ± 40; Molt4 = 130 ± 60; Jurkat = 160 ± 60; CCRF-CEM = 130 ± 20 (expressed as mean ± standard deviation).

### TS mRNA

A threefold variation in the TS mRNA/18S rRNA ratio was found between the human leukaemia cell lines (Figure 2A). The highest expression of TS mRNA relative to 18s rRNA was found in the CCRF-CEM cell line, and the lowest in the HL-60 cell line. However, RNA extracted from the HL-60 cell line was consistently found to be degraded (Figure 2A). Similarly, for the colorectal carcinoma cell lines, a twofold variation in TS mRNA/18 s rRNA was found, with the highest TS mRNA levels being found in the SW48, SW480, SW620 and HCT116 cell lines, and the lowest expression found in LoVo and Colo205 cells (Figure 2B).

© Cancer Research Campaign 1997

**Figure 2** Northern blot hybridization of TS mRNA for the human leukaemia (**A**) and human colorectal (**B**) cell lines. Each lane contained 20 μg of total RNA for the hybridization reaction to the 0.7-kb fragment of the pMTS-3 plasmid. The HL60 cell line consistently showed partial degradation of TS mRNA

**Figure 3** Relationship between TS activity and TS protein for the human leukaemia and human colorectal cell lines. □, Colorectal cell lines; ◆, leukaemic cell lines

**Figure 4** Relationship between the mean THYMITAQ™ $IC_{50}$ values and mean TS activity for the human colorectal and human leukaemic cell lines. □, Colorectal cell lines; ◆, leukaemic cell lines

### Relationships between TS activity, protein and mRNA and the growth-inhibitory activity of THYMITAQ™

When the measurements for the colorectal and leukaemic cell line panels were combined, a highly significant positive linear correlation ($r^2 = 0.87$, $P = 0.00002$) was found between TS protein and TS activity (Figure 3). For the colorectal cell lines alone, a positive linear correlation was also found between TS protein and TS activity ($r^2 = 0.48$, $P = 0.08$), but this did not reach statistical significance at the 5% level. Neither TS activity or TS protein was found to relate to TS mRNA/18s rRNA ratios as determined using the Kendall Rank correlation test, with either separate or pooled data for the colorectal and human leukaemia cell lines.

When analysed separately, there was no significant linear relationship between TS activity and THYMITAQ™ $IC_{50}$ for either colorectal ($r^2 = 0.4$, $P = 0.13$) or human leukaemia ($r^2 = 0.29$, $P = 0.34$) cell lines. In the case of the colorectal cell lines, a significant linear relationship is prevented by the relatively high TS protein level found in the LoVo cell line, which was relatively sensitive to THYMITAQ™. Similarly, when the data for the two cell line panels were combined, TS activity did not correlate with THYMITAQ™ $IC_{50}$ ($r^2 = 0.01$, $P = 0.69$). The lack of a relationship is due to the presence of two colorectal cell lines (BE and HCT 116) and one human leukaemic cell line (HL60) that were insensitive to THYMITAQ™ ($IC_{50} > 2$ μM), despite having relatively low levels of TS activity (Figure 4). When the results for these three THYMITAQ™-insensitive cell lines were excluded from the analysis, a significant relationship was found between THYMITAQ™ $IC_{50}$ and TS activity ($r^2 = 0.72$, $P = 0.005$) when the two cell line panels were combined.

There was no significant linear relationship between TS protein and THYMITAQ™ $IC_{50}$ for either the colorectal cell line panel ($r^2 = 0.36$, $P = 0.11$) or the leukaemic cell line panel ($r^2 = 0.38$, $P = 0.37$). Similarly, there was no correlation between TS mRNA expression and THYMITAQ™ $IC_{50}$ ($P > 0.5$) for either the colorectal or leukaemic cell lines. Investigation of potential relationships using a non-parametric method (Kendall Rank correlation analysis) produced similar conclusions to the statistical analysis using the above parametric tests.

### DISCUSSION

The aim of this study was to characterize intrinsic TS expression in a panel of human colorectal and leukaemia cell lines to investigate the relationship between TS activity, protein levels and mRNA, and the growth-inhibitory activity of THYMITAQ™, a specific non-classical antifolate TS inhibitor.

Previous studies using both colorectal and lymphoblastic cell lines with acquired resistance to TS inhibitors have demonstrated an associated overexpression of TS. Copur et al (1995) described the development of 5-FU resistance within the H630 cell line, in which tenfold resistance to 5-FU was associated with a 23-fold increase in TS activity and TS protein levels, and an 18-fold increase in TS mRNA/β-actin RNA ratio. Similarly, the development of acquired resistance to Tomudex has been characterized in a variety of cell lines, including the lymphoblastic cell line W1-L2 and the human ovarian carcinoma cell line CH1 (Freemantle et al,

1995; Jackman et al, 1995). For the W1-L2 cell line, resistance to Tomudex was associated with a 514-fold increase in TS activity, a 180-fold increase in TS protein levels and a 128-fold increase in the TS mRNA/18S rRNA ratio. TS overexpression was less pronounced in the CH1 cell line, in which a fourfold increase in TS activity, twofold increase in TS protein and, possibly, a twofold increase in TS mRNA levels was demonstrated. In both cell lines, defects in polyglutamation were also observed, emphasizing the potential importance of the latter as a mechanism of resistance to classical antifolate TS inhibitors.

The finding in the studies reported here, i.e. that, across all of the cell lines studied, there was a highly significant correlation between TS activity and TS protein as measured by quantitative Western blotting, is in keeping with the relationships reported by Jackman et al (1995). Moreover, the finding of higher TS activity and TS protein levels in the leukaemic cell lines is consistent with another report comparing the TS activities of haematological and colorectal cell lines, in which the human lymphoblastoid cell line W1-L2 was found to have a 10- to 20-fold higher TS activity than a panel of human colorectal carcinoma cell lines (Van der Wilt et al, 1993).

In the studies reported here, sensitivity to THYMITAQ™ within the panel of colorectal cell lines did not correlate with cellular TS protein content, for which the 20-fold range in THYMITAQ™ $IC_{50}$ values was related only to a fourfold variation in TS protein levels. This lack of a statistically significant correlation may have resulted both from the relative lack of sensitivity of the methodology used in the present study for determining small differences in TS protein expression, and from the finding of a relatively high TS protein content for the THYMITAQ™-sensitive LoVo cell line. A similar lack of a relationship between TS protein expression and sensitivity to TS inhibition by 5-fluorouracil has also been reported for human colorectal carcinoma cell lines (Berger and Berges, 1988).

Although in the present study the correlation between TS activity and THYMITAQ™ $IC_{50}$ did not reach statistical significance, this was largely because of the BE cell line. In BE cells, there was only moderate TS activity, which would not have been expected on the basis of the relatively high TS protein levels, and yet lack of sensitivity to THYMITAQ™. Also, in contrast to the results from one clinical study with 5-FU (Johnston et al, 1995), there was no correlation between sensitivity to THYMITAQ™ and TS mRNA expression in the colorectal cell lines. For the human leukaemia cell lines, a smaller range of TS expression was seen, and again no significant correlations were found between TS mRNA levels and sensitivity to THYMITAQ™.

In the present study, two of the colorectal adenocarcinoma cell lines (HCT116 and BE) and the promyelocytic HL60 cell line were found to display reduced sensitivity to THYMITAQ™, which would not have been predicted solely on the basis of their TS expression. When these cell lines were excluded from the analyses, a significant relationship between both TS activity and TS protein levels and the sensitivity to THYMITAQ™-mediated growth inhibition was found when the results of the colorectal and leukaemic cell line panels were combined. This finding suggests that factors other than TS expression can play an important role in determining the sensitivity of cell lines to THYMITAQ™. As THYMITAQ™ does not require active or facilitated transport and is not a substrate for polyglutamation, other factors must underline the differential sensitivity of the cell lines. Specifically, these factors may include cellular biochemical events 'downstream' of TS inhibition. For example, an increased capacity to repair DNA damage (Canman et al, 1992) and bcl-2-mediated tolerance to apoptotic stimuli (Fisher et al, 1993) have been reported to cause variations in the sensitivity of colorectal cell lines to both 5-fluorouracil and antifolate TS inhibition. These factors may also have contributed to the lack of a significant correlation between measures of TS expression found in the colorectal cell line panel in the present study and of sensitivity to THYMITAQ™.

Resistance to TS inhibition resulting from mutation of the target enzyme has also been reported, with Berger et al (1988) finding reduced sensitivity to 5-FU in a HCT116 cell line, which resulted from a novel, more basic charge form of TS. Specifically, the mutation was found to result from a tyrosine to histidine replacement at residue 33 of TS (Barbour et al, 1992). It is not currently known whether or not this or a similar mutation is present in the HCT116 cells used in the present study; and, if the mutation is present, the impact of this mutation on THYMITAQ™ sensitivity is also not known.

Neither TS protein nor TS activity were found to correlate with the TS mRNA/18S rRNA ratio in either the colorectal adenocarcinoma or leukaemic cell line panels. The lack of a relationship may possibly be explained by the finding that TS protein can regulate its own transcription (Chu et al, 1991), with protein levels not being a simple function of transcript concentration. Variation in this feedback mechanism may exist between different cell lines. Therefore, measurement of TSmRNA in a heterogenous population of cell lines may be a poor predictor of sensitivity to TS inhibition.

In summary, neither the colorectal or the leukaemia cell line panels demonstrated a significant correlation between measures of TS expression and sensitivity to THYMITAQ™-mediated growth inhibition. THYMITAQ™ resistance, which would not have been predicted solely on the basis of TS expression, was demonstrated in two of the colorectal and one of the leukaemia cell lines, suggesting that events downstream of TS inhibition can be important determinants of the growth-inhibitory activity of THYMITAQ™. There were no correlations between TS activity or TS protein and TS mRNA/18s rRNA ratios in either of the cell line panels, suggesting that TS expression as measured by TS mRNA alone may not be predictive of sensitivity to THYMITAQ™. Future studies will examine the prognostic significance of TS expression for response to THYMITAQ™ in adult patients with colorectal carcinoma and in children with acute lymphoblastic leukaemia at both presentation and relapse.

### ACKNOWLEDGEMENTS

This work was kindly supported by The Newcastle University Hospitals Special Trustees, The North of England Childrens Cancer Research Fund (EJE), Agouron Pharmaceuticals (KB), the Leukaemia Research Fund (AGH) and the North of England Cancer Research Campaign.

### REFERENCES

Barbour KW, Hoganson DK, Berger SH and Berger FG (1992) A naturally occurring tyrosine to histidine replacement at residue 33 of human thymidylate synthase confers resistance to 5-fluoro-2′-deoxyuridine in mammalian and bacterial cells. *Mol Pharmacol* **42:** 242–248

Berger SH and Berger FG (1988). Thymidylate synthase as a determinant of 5-fluoro-2-deoxyuridine response in human colonic tumour cell lines. *Mol Pharmacol* **34:** 474–479

© Cancer Research Campaign 1997

Berger SH, Barbour KW and Berger FG (1988) A naturally occurring variation in thymidylate synthase structure is associated with a reduced response to 5-fluoro-2′-deoxyuridine in a human colon tumour cell line. *Mol Pharmacol* **34:** 480–484

Calvert AH, Jones TR, Dady PJ, Grzelakowska-Sztabert B, Paine RM, Taylor GA and Harrap KR (1980) Quinazoline antifolates with dual biochemical loci of action. Biochemical and biological studies directed towards overcoming methotrexate resistance. *Eur J Cancer* **16:** 713–722

Canman CE, Tang HY, Normale DP, Lawrence TS and Maybaum J (1992) Variations in patterns of DNA damage induced in human colorectal cells by 5-fluorodeoxyuridine: implications for mechanisms of resistance and cytotoxicity. *Proc Natl Acad Sci USA* **89:** 10474–10478

Chomcyznski P and Sacchi N (1987) Single step method of RNA isolation by acid guanidinium thiocyanate–phenol–chloroform extract. *Anal Biochem* **162:** 156–159

Chu E, Koeller DM, Casey JL, Drake JC, Chabner BA, Elwood PC, Sydelle Z and Allegra CJ (1991) Autoregulation of human thymidylate messenger RNA translation by thymidylate synthase. *Proc Natl Acad Sci USA* **88:** 8977–8981

Copur S, Aiba K, Drake JC, Allegra CJ and Chu E (1995) Thymidylate synthase gene amplification in human colon cancer cell lines resistant to 5-fluorouracil. *Biochem Pharmacol* **49:** 1419–1426

Feinberg AP and Vogelstein B (1983) A technique for radiolabelling DNA restriction endonuclease fragments to high specific activity. *Anal Biochem* **132:** 6–13

Fisher TC, Milner AE, Gregory CD, Jackman AL, Aherne W, Hartley JA, Dive C and Hickman J (1993) bcl-2 modulation of apoptosis induced by anticancer drugs: resistance to thymidylate stress in independent of classical resistance pathways. *Cancer Res* **53:** 3321–3326

Freemantle SJ, Aherne GW, Hardcastle A, Lunec J and Calvert AH (1991) Increases in thymidylate synthase protein levels measured using newly developed antibodies. *Proc Am Assoc Cancer Res* **32:** 360

Freemantle SJ, Jackman AL, Kelland LR, Calvert AH and Lunec J (1995) Molecular characterisation of two cell lines selected for resistance to the folate-based thymidylate synthase inhibitor, ZD1694. *Br J Cancer* **71:** 925–930

Geyer PK and Johnson LF (1984) Molecular cloning of DNA sequences complementary to mouse thymidylate synthase messenger RNA. *J Biol Chem* **259:** 7206–7211

Jackman AL and Calvert AH (1995) Folate-based inhibitors as anticancer drugs. *Ann Oncol* **6:** 871–881

Jackman AL, Taylor GA, Gibson W, Kimbell R, Brown M, Calvert AH, Judson IR and Hughes IR (1991) ICID1694, a quinazoline antifolate thymidylate synthase inhibitor that is a potent inhibitor of L1210 tumour cell growth in vitro and in vivo: a new agent for clinical study. *Cancer Res* **51:** 5579–5586

Jackman AL, Kelland LR, Kimbell R, Brown M, Gibson W, Aherne GW, Hardcastle A and Boyle FT (1995) Mechanisms of acquired resistance to the quinazoline thymidylate synthase inhibitor ZD1694 (Tomudex) in one mouse and three human cell lines. *Br J Cancer* **71:** 914–924

Jansen G, Schnorgel JH, Westerhof GR, Rijksen G, Newell DR and Jackman AL (1990) Multiple membrane transport systems for the uptake of folate based thymidylate synthase inhibitors. *Cancer Res* **50:** 7544–7548

Johnston PG, Drake JC, Steinberg SM and Allegra C (1993) Quantitation of thymidylate synthase in human tumors using an ultrasensitive enzyme-linked immunoassay. *Biochem Pharmacol* **45:** 2483–2486

Johnston PG, Fisher ER, Rockette HE, Fisher B, Wolmark N, Drake JC, Chabner BA and Allegra CJ (1994) The role of thymidylate synthase expression in the prognosis and outcome of adjuvant chemotherapy in patients with rectal cancer. *J Clin Oncol* **12:** 2640–2647

Johnston PG, Lenz H-J, Leichman CG, Danenberg KD, Allegra CJ, Danenberg PV and Leichman L (1995) Thymidylate synthase gene and protein expression correlate and are associated with response to 5-fluorouracil in human colorectal and gastric tumours. *Cancer Res* **55:** 1407–1412

O'Connor BM, Jackman AL, Crossley PH, Freemantle SE, Lunec J and Calvert AH (1992) Human lymphoblastoid cells with acquired resistance to $C^2$-desamino-$C^2$-methyl $N^{10}$-propargyl-5,8-dideazafolic acid: a novel folate based thymidylate synthase inhibitor. *Cancer Res* **52:** 1137–1143

Peters GJ, Laurrensse E, Lankelma J, Leyva A and Pinedo HM (1986) Sensitivity of human, murine and rat cells to 5-fluorouracil and 5′ deoxy-5-fluorouridine in relation to drug metabolising enzymes. *Cancer Res* **46:** 20–28

Peters GJ, Van Groeningen CJ, Laurensse EJ and Pinedo HM (1991) Thymidylate synthase from untreated human colorectal cancer and colonic mucosa: enzyme activity and inhibition by 5-fluoro-2′-deoxyuridine-5′-monophosphate. *Eur J Cancer* **27:** 263–267

Pinedo HM and Peters GF (1988) Fluorouracil: biochemistry and pharmacology. *J Clin Oncol* **6:** 1653–1664

Rhee MS, Wang Y, Gopal Nair M and Galivan J (1993) Acquisition of resistance to antifolates caused by γ-glutamyl hydrolase activity. *Cancer Res* **53:** 2227–2230

Taylor GA, Jackman AL, Balmanno K, Hughes LR and Calvert AH (1988) Estimation of the *in-vitro* and *in-vivo* inhibitory effects of antifolates upon thymidylate synthase in whole cells. In *Purine Metabolism in Man VI* K Mikanagnagi K, Nifhioka K and Kelly WN. (eds), pp. 383–388. Plenum Press: New York

Touroutoglu N and Pazdur R (1996) Thymidylate synthase inhibitors. *Clin Cancer Res* **2:** 227–243

Van der Wilt CL, Smid K, Aherne GW, Pinedo HM and Peters GJ (1993) Evaluation of immunohistochemical staining and activity of thymidylate synthase in cell lines. In *Chemistry and Biology of Pteridines and Folates*, Ayling JE et al. (eds), pp. 605–608. Plenum Press: New York

Webber SE, Bleckman TH, Attard J, Deal JG, Kathardekar V, Welsh KM, Webber S, Janson CA, Matthews DA, Smith WW, Freer ST, Jordan SR, Baquet RJ, Howland EF, Booth CLJ, Ward RW, Herrman SM, White J, Morse CA, Hilliard JA and Bartlett CA (1993) Design of thymidylate synthase inhibitors using protein crystal structures: the synthesis and biological evaluation of a novel class of 5-substituted quinazolinones. *J Med Chem* **36:** 733–746

Webber S, Barlett CA, Boritzki TJ, Hilliard JA, Howland EF, Johnston AL, Kosa, M, Margosiak SA, Morse CA and Shetty BV (1996) THYMITAQ™, a novel lipophilic thymidylate synthase inhibitor: *in vitro* and *in vivo* preclinical studies. *Cancer Chemother Pharmacol* **37:** 509–517

© Cancer Research Campaign 1997